# R-loop formation by dCas9 is mutagenic in *Saccharomyces cerevisiae*

**DOI:** 10.1093/nar/gky1278

**Published:** 2018-12-21

**Authors:** Marian F Laughery, Hannah C Mayes, Ivan K Pedroza, John J Wyrick

**Affiliations:** 1 School of Molecular Biosciences, Washington State University, Pullman, WA 99164, USA; 2 Center for Reproductive Biology, Washington State University, Pullman, WA 99164, USA

## Abstract

Cas9 binds and cleaves specific DNA sequences by inducing the formation of an R-loop between the guide RNA and its genomic target. While targeting of active Cas9 to a genomic locus is highly mutagenic because Cas9 creates DNA double strand breaks, targeting of dead Cas9 (dCas9) is presumed not to be mutagenic, as dCas9 lacks DNA endonuclease activity. Here, we show that dCas9 targeting induces mutations in yeast, particularly when targeted to the non-transcribed strand of a gene. dCas9-induced mutations cluster near the guide RNA target region and are comprised of single nucleotide substitutions, small insertions and deletions, and even complex mutations, depending upon the particular guide RNA target. We show that many of these mutations are a consequence of cytosine deamination events occurring on the non-target strand of the dCas9-induced R-loop, while others are associated with homopolymer instability or translesion DNA synthesis. Targeting of dCas9 by a mismatch-containing guide RNA also increases *CAN1* mutation frequency, particularly in an *ung1*Δ mutant strain, suggesting that dCas9 induces mutations through similar mechanisms at off-target sites. These findings indicate that DNA binding by dCas9 is mutagenic in yeast, likely because dCas9 induces the formation of an R-loop at its target site.

## INTRODUCTION

CRISPR/Cas9 RNA-guided endonucleases are a transformative tool for eukaryotic genome engineering (1,2) due to their unique ability to bind and cleave specific DNA sequences that are complementary to a Cas9-bound single-guide RNA (sgRNA). Initial applications of Cas9 for genome engineering relied on its ability to generate targeted DNA double strand breaks (DSBs), which induce DSB repair pathways that can introduce designed or random mutations at the break site. Subsequent applications have utilized an enzymatically ‘dead' mutant of Cas9 (dCas9) to target Cas9-fused ‘cargos', such as transcriptional activation or repression domains (3–8), histone modifying enzymes (9–11) or cytidine deaminases (12–14), to specific genomic locations. Specific targeting of Cas9 (or dCas9) relies on the formation of an ∼20-nucleotide-long R-loop, consisting of an RNA:DNA hybrid between the target DNA strand and the Cas9-bound sgRNA (15). R-loop formation is initiated upon Cas9 binding to a protospacer adjacent motif (PAM) sequence, which for *Streptococcus pyogenes* Cas9 consists of a 5′-NGG-3′ sequence (16,17). Following PAM recognition, Cas9 promotes the progressive unwinding of the DNA target and sgRNA invasion, beginning with the ∼10 nucleotide ‘seed' region of the sgRNA that is immediately proximal to the PAM (17). This results in the formation of an R-loop between the target DNA strand and the sgRNA, which induces a conformational change in the Cas9 protein that triggers DNA cleavage (18,19).

It is well known that Cas9 can bind and cleave many off-target sites (20,21), as Cas9 tolerates sgRNA–DNA mismatches, particularly outside of the critical sgRNA seed region. These off-target cleavage events can induce background mutations when repaired by the error-prone non-homologous end joining or alternative end joining pathways. In addition, point mutations are occasionally generated during Cas9 genome editing in mammalian cells. These point mutations have been attributed to the activity of apolipoprotein B mRNA editing enzyme, catalytic polypeptide-like (APOBEC) cytidine deaminases, which deaminate cytosine residues in single-stranded DNA intermediates generated during repair of Cas9-induced double-strand or single-strand breaks (22,23). The specificity of Cas9 DNA binding is generally less stringent than the specificity of Cas9-induced DNA cleavage (20,24,25), since Cas9 binding does not require extended R-loop formation and can tolerate additional sgRNA–DNA mismatches. However, Cas9 binding alone, whether at off-target sites that are not cleaved or on-target sites bound by dCas9, has been generally assumed to not be mutagenic.

R-loops are not only generated during Cas9 genome editing, but occur frequently in cellular DNA, particularly at actively transcribed genes. Endogenous R-loops are thought to form when a nascent mRNA threads back to reanneal with the transcribed DNA strand in the wake of an elongating RNA polymerase (26,27). Recent evidence suggests that R-loops induce genome instability by stimulating DNA recombination and mutagenesis (26). In *Escherichia coli*, transcription-associated mutagenesis is primarily elevated on the non-transcribed strand (NTS) of a gene (28,29), likely because the NTS adopts a single-stranded DNA (ssDNA) conformation when the nascent mRNA hybridizes with the transcribed strand (TS) to form an R-loop (26). ssDNA is particularly vulnerable to DNA damaging agents and is associated with elevated rates of spontaneous cytosine deamination and depurination events (26,28,30). R-loops can also stimulate mutagenesis by causing replication stress, due to stalling of the DNA replication machinery at R-loop containing DNA (27,31). While the formation of endogenous R-loops during transcription is associated with genome instability and mutagenesis, it is not known whether R-loops generated during Cas9 (or dCas9) targeting are also mutagenic.

Here, we show that targeting of dCas9 to the NTS of the yeast *CAN1* gene is mutagenic in yeast. Three different guide RNAs targeting different sites in the NTS increase *CAN1* mutation frequencies by up to ∼100-fold. Guide RNAs targeting the TS also induce *CAN1* mutagenesis, but to a somewhat lesser extent. dCas9 causes a biased mutation spectrum at multiple target sites that is consistent with elevated rates of cytosine deamination of the non-target DNA strand in the dCas9-induced R-loop. We also observe an elevated frequency of small insertion and deletion events and Rev3-dependent complex mutations at certain guide RNA targets, suggesting that dCas9 R-loop formation may promote replication stress. Targeting dCas9 with a mismatch-containing guide RNA also induces *CAN1* mutagenesis, indicating that dCas9 binding to off-target sites is also mutagenic.

## MATERIALS AND METHODS

### Yeast strains and plasmids

The wild-type (WT) yeast strain is derived from BY4741 (*MATa his3Δ1 leu2Δ0 met15Δ0 ura3Δ0*). The *ung1*Δ mutant was made in yeast strain MP019 (BY4741 + *trp1*Δ::*HIS3*) using a *TRP1* knockout construct. The p415-GalL-Cas9-CYC1t (i.e. pGAL-Cas9) expression vector (Addgene #43804) has been previously described (32). Stepwise site-directed mutagenesis was performed using a modified version of the QuikChange protocol (33) to construct D10A and H840A mutations in Cas9, yielding the pGAL-dCas9 expression plasmid. Plasmids encoding sgRNAs were constructed by ligation of hybridized oligonucleotides into the cut pTO40 vector, as previously described (34). Correct guide RNA insertion into the pTO40 expression vector was confirmed by sequencing using the T3 primer.

### Assessing *CAN1* mutation frequency

Yeast cells containing the dCas9 and sgRNA (or empty control) vectors were grown in synthetic complete (SC) media lacking uracil and leucine (SC-Ura-Leu) in order to maintain selection for the dCas9 and sgRNA expression vectors, diluted with water or phosphate buffered saline (PBS), and plated on SC-Ura-Leu plates containing galactose to induce pGAL-dCas9 expression. Plates were incubated at 30°C until large, isolated colonies appeared, typically 5–6 days. Five or six single colonies were individually resuspended and diluted in PBS. Appropriate dilutions were typically plated to both synthetic complete (SC) media (to determine the number of cells plated) and SC-Arg + 0.006% (w/v) canavanine (to assess the frequency of Can^R^ mutants). Plates were incubated at 30°C for about 3–5 days before counting colonies. A minimum of two replicates was performed for each sgRNA experiment.

Mutation frequencies were determined by calculating the ratio of the number of cells acquiring canavanine resistance to the number of cells that are viable in the absence of canavanine selection, normalized by the corresponding dilution factors (see formula below), as previously described (35,36).

\begin{equation*}{\rm Mutation}\ {\rm Freq.}\ = \ \frac{{\left( {\# \ {\rm of}\ {{\rm Can}^{\rm R}}\ {\rm colonies}} \right)\left( {{\rm Dilution}\ {\rm for}\ {\rm Canavanine}\ {\rm plates}} \right)}}{{\left( {\# \ {\rm of}\ {\rm colonies}\ {\rm on}\ {\rm SC}\ {\rm plate}} \right)\left( {{\rm Dilution}\ {\rm for}\ {\rm SC}\ {\rm plates}} \right)}}\end{equation*}

### Analyzing *CAN1* mutation spectra

Yeast cells were grown and plated on SC-Ura-Leu media containing galactose as described above. Colonies were then either replica plated or struck for isolation to SC-Arg + 0.006% Canavanine plates and incubated at 30°C long enough for colonies to grow, typically 3–4 days. Colonies were then struck for isolation or patched one or two more times to SC-Arg + 0.006% Canavanine plates. Genomic DNA (gDNA) was isolated by growing cells in YPD medium, harvesting cells, and vortexing with glass beads in DNA lysis buffer (2% Triton X-100, 1% SDS, 100 mM NaCl, 10 mM Tris–HCl pH 7.5, 1 mM EDTA). The genomic DNA was purified by phenol:chloroform:isoamyl alcohol (PCI) extraction, ethanol precipitated, and then digested with RNase A at 37°C for ∼30 min.

The *CAN1* locus was PCR amplified from gDNAs using Phusion HF DNA polymerase (NEB) and flanking primers OTM92 (5′-TATGAGGGTGAGAATGCGAAATGGCG-3′) and OTM93 (5′-AAGAGTGGTTGCGAACAGAGTAAACC-3′). PCR products were purified using the DNA Clean & Concentrator-5 Kit (Zymo Research) and were sequenced by either by Genscript or the WSU Molecular Biology and Genomics Core Facility. *CAN1* sequencing primers are available upon request. A complete list of identified *can1* mutations are compiled in Supplementary Table S1.

## RESULTS

### Targeting of dCas9 to the non-transcribed strand induces mutagenesis in yeast

**Figure 1.**
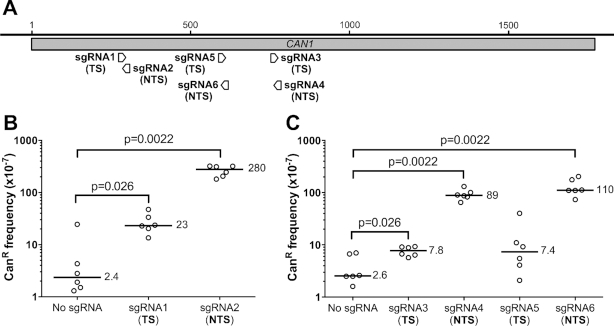
Targeting dCas9 to the yeast *CAN1* gene induces mutagenesis. (**A**) Schematic showing the target sites of sgRNA1, sgRNA3, and sgRNA5, which target the *CAN1* transcribed strand (TS), and sgRNA2, sgRNA4, and sgRNA6, which target the *CAN1* non-transcribed strand (NTS). The tip of the arrow indicates the PAM site for each sgRNA target. (**B**) Frequencies of canavanine-resistant (Can^R^) mutants in WT yeast (BY4741) expressing dCas9 (under the control of pGAL promoter) and the indicated guide RNA. Yeast strains were typically grown for ∼6 days on galactose-containing plates to induce dCas9 expression, and then plated on glucose-containing plates with and without canavanine to calculate the Can^R^ mutation frequency, as described in the Methods section. The median frequency of Can^R^ mutants is given for each strain. Statistical significance was calculated using a two-tailed Mann–Whitney U test. (**C**) Frequencies of canavanine-resistant (Can^R^) mutants in WT yeast (BY4741) expressing dCas9 and the indicated guide RNA (sgRNA3–6), as described in part B. Statistical significance was calculated using a two-tailed Mann–Whitney U test.

We hypothesized that dCas9 binding could stimulate mutagenesis *in vivo*, since dCas9 induces the formation of a ∼20-nucleotide R-loop that is potentially mutagenic. To test this hypothesis, we targeted dCas9 to the yeast *CAN1* locus using an sgRNA complementary to either the transcribed strand (TS) or non-transcribed strand (NTS) of *CAN1* (sgRNA1 and sgRNA2 in Figure 1A). Yeast cells expressing dCas9 and guide RNA were plated on canavanine-containing media to isolate loss-of-function mutations in *CAN1*, which render yeast resistant to canavanine (Can^R^). While the guide RNAs were constitutively expressed, expression of dCas9 was under the control of the pGAL promoter (32). dCas9 expression was induced by growth on galactose-containing plates for ∼6 days prior to assaying for *CAN1* mutations on glucose medium with canavanine. This strategy was used to repress dCas9 expression during selection for Can^R^ cells on canavanine-containing plates, since dCas9 binding has been previously shown to inhibit transcription in *Escherichia coli* (37), which could interfere with selecting for Can^R^ mutants.

Targeting of dCas9 to *CAN1* in yeast by either sgRNA1 or sgRNA2 caused a significant increase in the frequency of Can^R^ mutants (Figure 1B). This effect was most apparent for sgRNA2, which targets the *CAN1 NTS* at a location ∼290 bp downstream of the gene start. The Can^R^ frequency for dCas9/sgRNA2 expressing yeast was up to ∼100 fold higher than the no guide RNA control (*P* = 0.0022). Targeting dCas9 to the *CAN1* TS (sgRNA1) also significantly increased Can^R^ frequency (*P* = 0.026). However, the magnitude of this increase (∼10-fold relative to the ‘No sgRNA' control in Figure 1D) was significantly lower than when the NTS was targeted (*P* = 0.0022). These results indicate that dCas9 targeting significantly stimulates mutagenesis, particularly when the dCas9/guide RNA complex is targeted to the *CAN1* NTS.

To assess the generality of this conclusion, we tested additional guide RNAs (sgRNA3-sgRNA6) targeting the TS and NTS at different locations in *CAN1* (Figure 1A). We found that guide RNAs targeting the NTS consistently induced a significantly higher frequency of Can^R^ colonies relative to the no guide RNA control (35-fold and 44-fold increase for sgRNA4 and sgRNA6, respectively, in Figure 1C). In contrast, Can^R^ frequency was modestly elevated by guide RNAs targeting the TS (i.e. sgRNA3 and sgRNA5; see Figure 1C). These data indicate that targeting of dCas9 to the NTS of *CAN1* is particularly mutagenic in yeast.

### dCas9-induced mutations cluster near the guide RNA target

**Figure 2.**
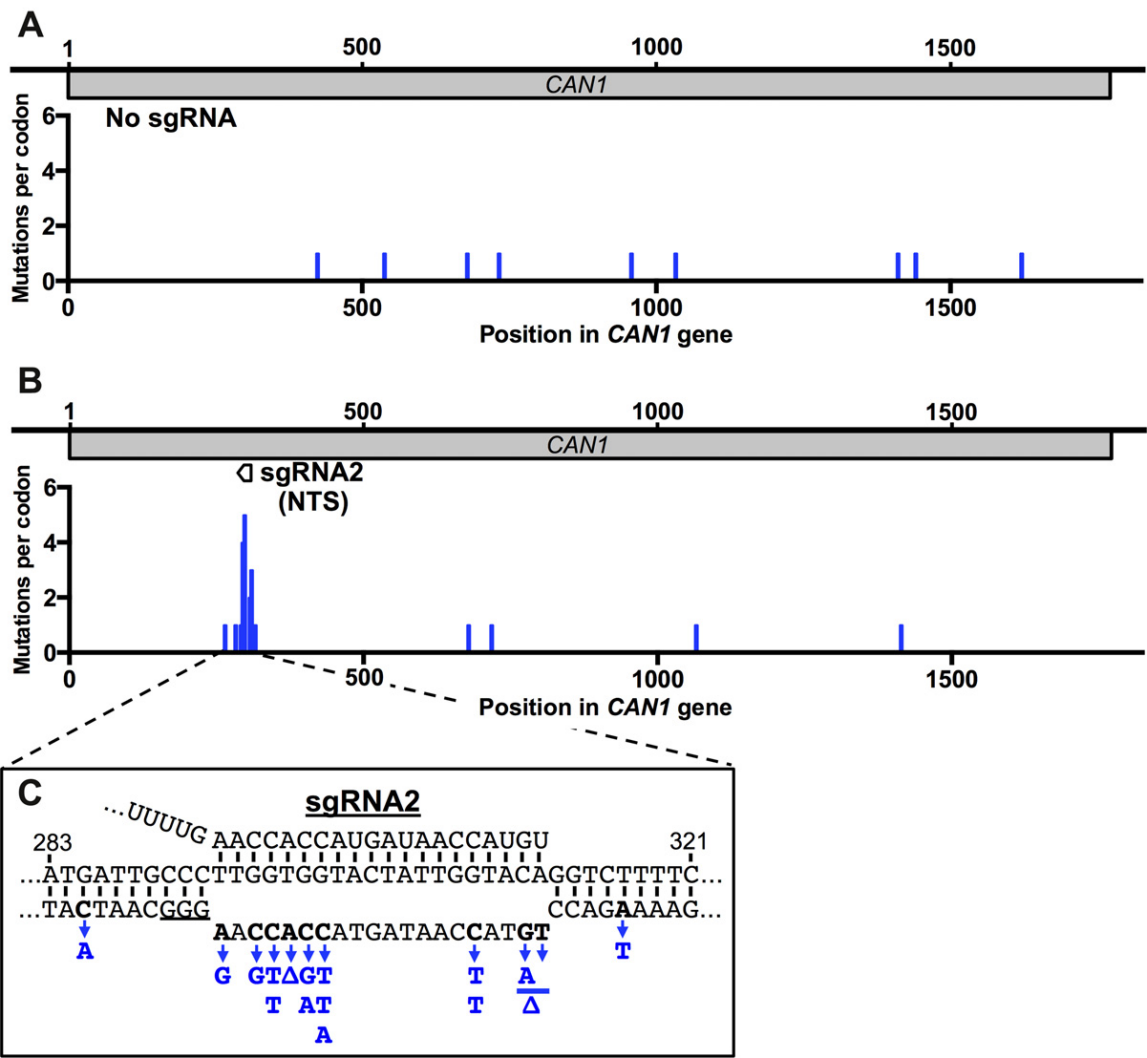
dCas9-induced *can1* mutations are clustered at the guide RNA target. (**A**) Distribution of spontaneous Can^R^ mutations in WT strain expressing dCas9 but no guide RNA. Mutations per codon are plotted. (**B**) Same as panel A, except for WT yeast expressing dCas9 and sgRNA2, which targets the *CAN1* non-transcribed strand (NTS). (**C**) dCas9/sgRNA2 primarily induce mutations at cytosine nucleotides on the non-target strand. The dCas9/sgRNA2-induced substitution mutations or deletions (Δ) are indicated in blue.

To investigate the mechanism of dCas9-induced mutagenesis, we PCR amplified and sequenced the *CAN1* locus from independent Can^R^ yeast colonies derived from a yeast strain expressing dCas9 along with either sgRNA2 (NTS) or no sgRNA. In the ‘No sgRNA' control, mutations were widely dispersed across the *CAN1* coding region (Figure 2A), consistent with these mutations occurring spontaneously throughout the *CAN1* gene. In the dCas9/sgRNA2 expressing strain, there was a striking cluster of mutations localized to the sgRNA2 target sequence (Figure 2B). Overall, 16 out of the 21 sgRNA2-derived Can^R^ mutants (76%) had a mutation within 10 nucleotides (nt) of the sgRNA2 target, and 14 of these were located directly inside the 20-nt sgRNA2 target sequence. These data indicate that dCas9-induced mutations cluster in the guide RNA target region.

To confirm these findings, we sequenced Can^R^ mutations derived from yeast expressing dCas9/sgRNA4, which targets the non-transcribed strand at a different location in the *CAN1* gene. We observed significant clustering of mutations near the guide RNA target (Supplementary Figure S1A), although to a lesser extent than was observed for sgRNA2. Overall, 7 out of 20 Can^R^ mutants (35%) were located within 2 nt of the sgRNA4 target. Interestingly, hotspots of sgRNA4-induced mutations occurred primarily on the periphery of the sgRNA4 target (i.e. 1 or 2 nts downstream of the guide RNA target, see Supplementary Figure S1B), unlike sgRNA2-induced mutations, which were enriched within the target sequence. While the differences in mutation distribution between the guide RNAs may reflect sequence constraints related to which DNA changes affect Can1 function, these data confirm that dCas9-induced mutations frequently cluster within or nearby the guide RNA target.

### Cytosine deamination in the dCas9-induced R-loop promotes mutagenesis

Closer inspection of the dCas9/sgRNA2-induced mutation spectrum revealed an enrichment of single nucleotide substitutions associated with cytosine nucleotides on the non-target DNA strand in the dCas9-induced R-loop. The 14 mutations in the sgRNA2 target sequence were primarily single nucleotide substitutions (Figure 2C), with most of these occurring at cytosine nucleotides in the non-target DNA strand (10 out of 14 mutations, 71%). This is likely not due to DNA sequence bias, as cytosine nucleotides comprise only 6 out of the 20 nts in the non-target DNA strand (30%). The most common substitution in the sgRNA2 target was C>T (6 out of 14), while C>G and C>A substitutions were the second most common (2 out of 14 each). In the sgRNA4-induced Can^R^ mutants, there were more deletions than substitutions in the sgRNA4 target, but the only substitution mutant in the target sequence was a C>G mutation (Supplementary Figure S1B).

**Figure 3.**
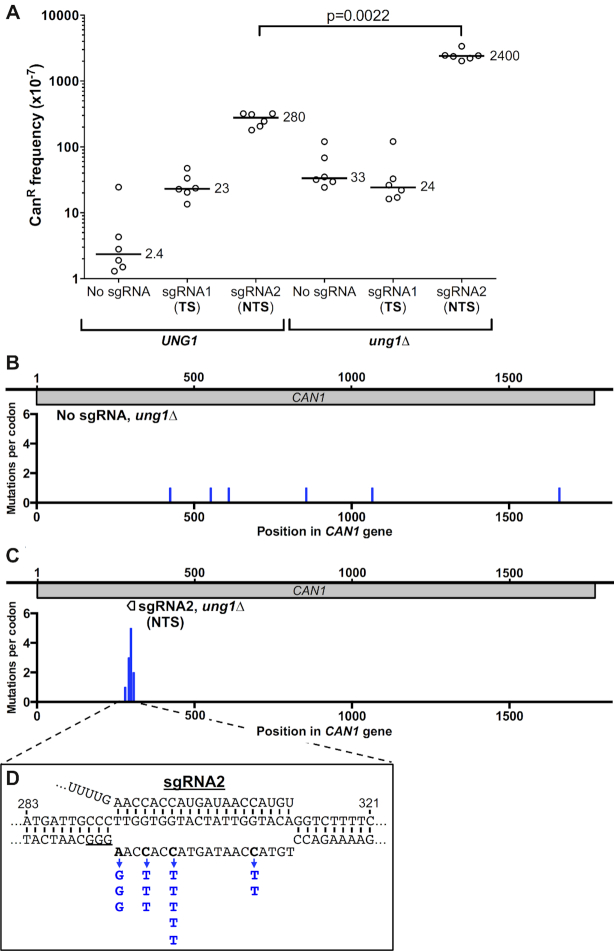
dCas9-induced mutagenesis is significantly elevated in an *ung1*Δ mutant strain when targeted to the NTS. (**A**) Frequency of canavanine-resistant (Can^R^) mutants in WT (*UNG1*) or *ung1*Δ mutant strains expressing dCas9 and the indicated guide RNA, as described in the Figure 1 legend. Data for *UNG1* wild-type strain is from Figure 1, and is included for reference. Statistical significance was calculated using a two-tailed Mann–Whitney U test. (**B**) Distribution of spontaneous Can^R^ mutations in *ung1*Δ strain expressing dCas9 but no guide RNA. Mutations per *CAN1* codon is plotted. (**C**) Same as panel B, except for an *ung1*Δ strain expressing dCas9 and sgRNA2. (**D**) dCas9/sgRNA2 primarily induce C>T mutations on the non-target strand in an *ung1*Δ mutant background, consistent with the model that dCas9 binding promotes cytosine deamination on the non-target DNA strand. The dCas9/sgRNA2-induced substitution mutations are indicated in blue.

The pattern of mutations within the sgRNA2 target suggests that many of these mutations may be derived from spontaneous cytosine deamination in the non-target DNA strand, as cytosine deamination typically causes C>T and C>G mutations, depending upon how the resulting uracil lesion is processed (36,38). To test this hypothesis, we measured dCas9-induced mutation frequencies in an *ung1*Δ mutant strain, since Ung1 (a uracil DNA glycosylase) normally suppresses mutagenesis due to cytosine deamination by initiating repair of the resulting uracil lesions. Deletion of *ung1* in the dCas9 and sgRNA2 expressing strain increased the frequency of Can^R^ mutants by ∼9-fold (Figure 3A) compared to the mutation frequency when expressing dCas9 and sgRNA2 in the WT control (*P* = 0.0022). This increase was not simply due to an increase in the background mutation rate, as Can^R^ mutation frequency was only marginally elevated in the *ung1*Δ strain when no sgRNA was expressed (Figure 3A). These results are consistent with the hypothesis that targeting of *CAN1* by dCas9 and sgRNA2 stimulates mutagenesis by inducing cytosine deamination in the non-target DNA strand.

To further test the hypothesis that dCas9/sgRNA2 promotes mutagenesis through a cytosine deamination mechanism, we sequenced the *CAN1* locus in Can^R^ mutants derived from an *ung1*Δ mutant strain expressing dCas9 and either ‘No sgRNA' (control) or sgRNA2. Since cytosine deamination in ssDNA primarily causes C>T and C>G mutations, with C>G (and presumably C>A) mutations caused by Ung1-mediated repair activity (38), deletion of *ung1* should enrich for C>T mutations in the non-target DNA strand. Can^R^ mutations were widely dispersed when no sgRNA was expressed in the *ung1*Δ mutant background (Figure 3B), but were clustered near the sgRNA2 target site when dCas9/sgRNA2 were expressed in *ung1*Δ strain (Figure 3C), as previously observed in WT cells (see Figure 2B). The mutation spectrum induced by dCas9/sgRNA2 in the *ung1*Δ mutant strain primarily consisted of C>T substitutions in the non-target strand (Figure 3D). Unlike WT cells, no C>G or C>A substitutions were detected in the non-target strand in the *ung1*Δ mutant background. There was also a hotspot of A>G mutations at a single location in the non-target strand (Figure 3D). Although the underlying mechanism for this mutation hotspot is unclear, these mutations could reflect spontaneous adenine depurination or deamination on the non-target strand (39). In general, the dCas9/sgRNA2-induced mutations in the *ung1*Δ mutant background occurred at the same locations as C>T (or A>G) mutations in the non-target strand in the WT background (i.e., position 293, 296, 299 and 308 in the *CAN1* coding region). Presumably, this reflects constraints related to which DNA sequence changes affect Can1 protein function. Taken together, these findings support our hypothesis that dCas9 targeting by sgRNA2 induces mutagenesis on the non-target DNA strand in part by stimulating cytosine deamination.

Cytosine deamination can occur spontaneously, particularly in ssDNA, or can be enzymatically induced by cytidine deaminases. While yeast lack canonical DNA cytidine deaminases, such as the AID and APOBEC family of enzymes found in higher eukaryotes (40), it has recently been reported that the Fcy1 cytosine deaminase in yeast can target R-loops to stimulate mutagenesis at CAG/CTG repeat sequences (41). To test whether Fcy1 is also required for mutagenesis at dCas9-induced R-loops, we measured the Can^R^ frequency following dCas9 targeting to *CAN1* in an *fcy1*Δ mutant strain. For sgRNA1/dCas9 and sgRNA2/dCas9 expressing strains, there was no significant difference in Can^R^ frequency in the *fcy1*Δ mutant background relative to WT (Supplementary Figure S2), indicating that dCas9-induced mutagenesis is not dependent on Fcy1 activity. These results indicate that mutagenic cytosine deamination events occurring on the non-target strand of the dCas9-induced R-loop likely occur spontaneously, presumably due to the higher reactivity of cytosines in ssDNA (30).

### dCas9 targeting to the *CAN1* TS causes complex mutations dependent upon DNA polymerase zeta

The frequency of the Can^R^ mutants was only marginally altered, if at all, when *ung1* was deleted in the dCas9/sgRNA1 expressing strain (Figure 3A). This result suggests that targeting of dCas9 to the *CAN1* TS by sgRNA1 induces mutagenesis through a distinct mechanism, not involving cytosine deamination. To investigate this mechanism, we sequenced the *CAN1* gene from independent Can^R^ isolates derived from the sgRNA1/dCas9 expressing strain. DNA sequencing revealed clustering of mutations near the sgRNA1 target site (nucleotides 277–296 in *CAN1*), with 9 of 19 mutations (47%) located in the sgRNA1 target (Table 1). Many of the mutations in the sgRNA1 target site were insertions or deletions, and 3 of the 9 target site mutations were complex mutations (Table 1), defined as two or more mutation events occurring within 10 nucleotides of each other (42).

**Table 1.** sgRNA1/dCas9-induced *can1* mutation spectrum

*can1* mutation	Complex mutation?
259G>T	
263T>G	
282T>A, 286–287del*	**+**
286–287del*	
287T>A*	
287T>A, 289G>C*	**+**
290C>A*	
290ins(22 bp)*	
291C>G, 294T>A*	**+**
292del*	
294ins(T)*	
591C>A	
801–808del	
809G>A	
892del	
923G>T	
973G>T	
1035C>A	
1068C>A	

*Mutation in sgRNA1 target sequence.

**Figure 4.**
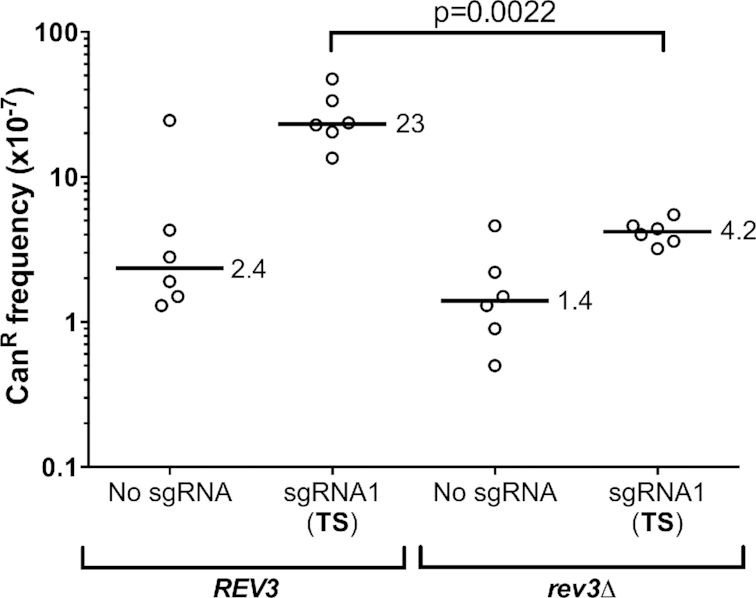
dCas9/sgRNA1-induced mutagenesis is dependent upon translesion DNA synthesis by DNA polymerase zeta (pol ζ). Frequency of canavanine-resistant (Can^R^) mutants in WT (*REV3*) or *rev3*Δ mutant (lacking pol ζ activity) expressing dCas9 and sgRNA1. The median frequency of Can^R^ mutants is given for each strain. Statistical significance was calculated using a two-tailed Mann–Whitney U test.

Complex mutation events are a signature of translesion DNA synthesis (TLS), particularly by DNA polymerase zeta (pol ζ, (43,44)). To test whether dCas9/sgRNA1-induced mutations were due to TLS activity, we measured the effect of a *rev3*Δ mutant, which inactivates pol ζ, on *CAN1* mutagenesis. The frequency of Can^R^ mutants induced by dCas9/sgRNA1 was significantly reduced in the *rev3*Δ mutant background (Figure 4), indicating that pol ζ is important for dCas9 to induce mutations at this target site. Taken together, these data indicate that targeting of dCas9 to the TS induces mutations through a distinct mechanism that is dependent upon TLS activity by DNA polymerase ζ.

### Other guide RNAs also promote cytosine deamination-mediated mutagenesis

Since dCas9 targeting causes only a 20 nucleotide R-loop, we wondered whether DNA sequence constraints in this relatively small R-loop forming region might limit which guide RNAs can induce loss-of-function *can1* mutations through a cytosine deamination mechanism. To test this, we analyzed a large dataset of sequenced *can1* mutants induced by APOBEC expression in yeast (36,45), since APOBEC enzymes induce mutations by catalyzing cytosine deamination. Based on this analysis, the number of potential *CAN1*-inactivating cytosines (i.e. cytosines which when mutated by a deamination event give rise to Can^R^) in the non-target strand of each guide RNA was determined, and ranged from 0 (sgRNA1 and sgRNA3) to 5 (sgRNA2; see Supplementary Table S2). dCas9-induced mutation frequency was correlated with the number of potential *CAN1*-inactivating cytosines in the non-target strand (compare Supplementary Table S2 with Figure 1). Notably, the guide RNAs targeting the TS generally had much fewer *CAN1*-inactivating cytosines on the non-target strand than guide RNAs targeting the NTS (Supplementary Table S2), which could potentially explain the lower Can^R^ frequencies for the TS guides.

**Figure 5.**
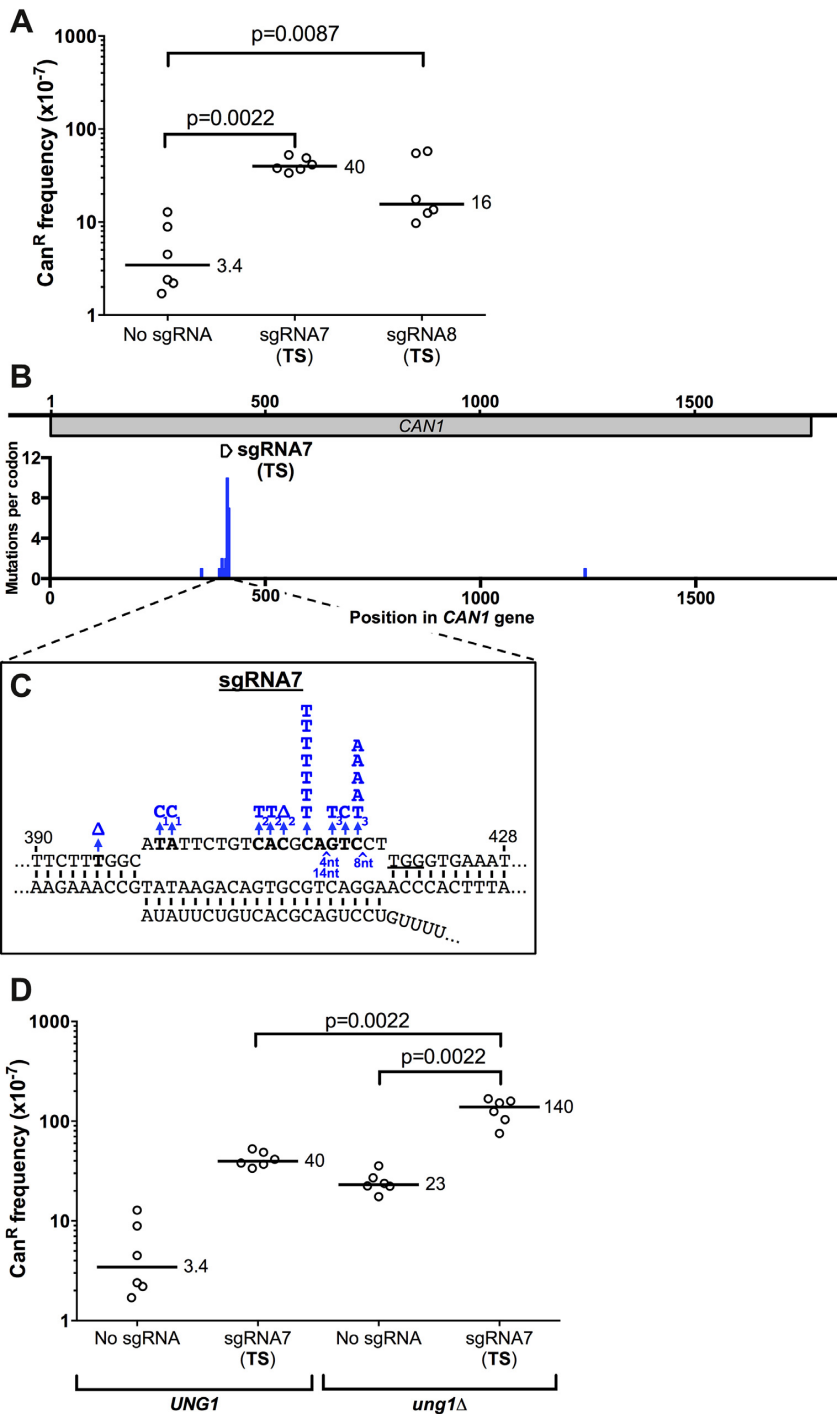
sgRNAs targeting the transcribed strand (TS) also significantly stimulate dCas9 mutagenesis through a cytosine deamination mechanism. (**A**) Frequency of canavanine-resistant (Can^R^) mutants in WT strain expressing dCas9 and the indicated guide RNA, as described in the Figure 1 legend. (**B**) Distribution of dCas9/sgRNA7-induced Can^R^ mutations. Mutations per *CAN1* codon is plotted. Location of sgRNA7 guide RNA target is depicted for reference. If multiple nucleotides are substituted or deleted, each mutated nucleotide is counted as an individual mutation. (**C**) dCas9/sgRNA7 primarily induce mutations at cytosine nucleotides on the non-target strand, particularly at C412 and C416. All mutations are indicated in blue, deletions are indicated with a Δ, and insertions are indicated with a ∧, along with the size of the insertion (i.e. number of nucleotides (nt) inserted). Each of the mutations in a complex mutation event is indicated with the same subscript (i.e. C_1_C_1_). (**D**) Frequency of Can^R^ mutations when dCas9/sgRNA7 are expressed in an *ung1*Δ mutant strain. Data for *UNG1* wild-type strain is from part A, and is included for reference. Statistical significance was calculated using a two-tailed Mann–Whitney U test.

To test this hypothesis, we designed two new guide RNAs, sgRNA7 and sgRNA8, which targeted overlapping regions of the TS of *CAN1* and contained three and two potential *CAN1*-inactivating cytosines, respectively (Supplementary Table S2). Expressing dCas9 with either sgRNA7 or sgRNA8 significantly increased the frequency of Can^R^ mutants relative to the no guide RNA control (Figure 5A). While the Can^R^ frequency induced by these new guides was similar to or greater than the previous TS guide RNAs, it was still significantly lower than the NTS guides (compare Figure 5A and Figure 1), even though sgRNA7 and sgRNA8 have a comparable number of *CAN1*-inactivating cytosines in their target sites as the NTS guide RNAs (Supplementary Table S2). These findings support our hypothesis that targeting the NTS with dCas9 is generally more mutagenic than targeting the TS.

Sequencing of Can^R^ colonies from dCas9/sgRNA7 expressing cells again revealed significant clustering of dCas9-induced mutations near the guide RNA target sequence (Figure 5B). Of the 21 sequenced Can^R^ isolates, 19 had mutations within or immediately adjacent to the sgRNA7 guide RNA target. There was enrichment of complex mutations (3 out of 19) and multiple nucleotide insertions (3 out of 19) in the sgRNA7 target site. Notably, these multiple nucleotide insertions create direct repeats in the guide RNA target site (Supplementary Table S1). These results are consistent with sequencing data for the dCas9/sgRNA1 expressing cells (Figure 5C and Supplementary Table S1), which also targets the TS, suggesting that these complex mutations may also be pol ζ-dependent.

There was also significant enrichment of substitution mutations at C nucleotides in the non-target strand (11 out of 19 mutations), particularly at hotspot sites at nucleotides C412 and C416 in the guide RNA target (Figure 5C). These mutations occur at sites predicted by our analysis to be potential *CAN1*-inactivating cytosines (Supplementary Table S2). This pattern in the mutation spectrum is consistent with dCas9/sgRNA7 promoting the deamination of cytosines in the non-target strand of the dCas9-induced R-loop, as previously observed for dCas9/sgRNA2 expressing cells. To further test whether cytosine deamination was important for mutagenesis in dCas9/sgRNA7 expressing cells, we measured the Can^R^ frequency when dCas9/sgRNA7 were expressed in an *ung1*Δ mutant strain. The Can^R^ mutation frequency was significantly elevated when dCas9/sgRNA7 were expressed in the *ung1*Δ mutant (Figure 5D), supporting the hypothesis that dCas9/sgRNA7 can induce mutations through a cytosine deamination mechanism.

### dCas9 binding stimulates mutagenesis in a homopolymer sequence

**Figure 6.**
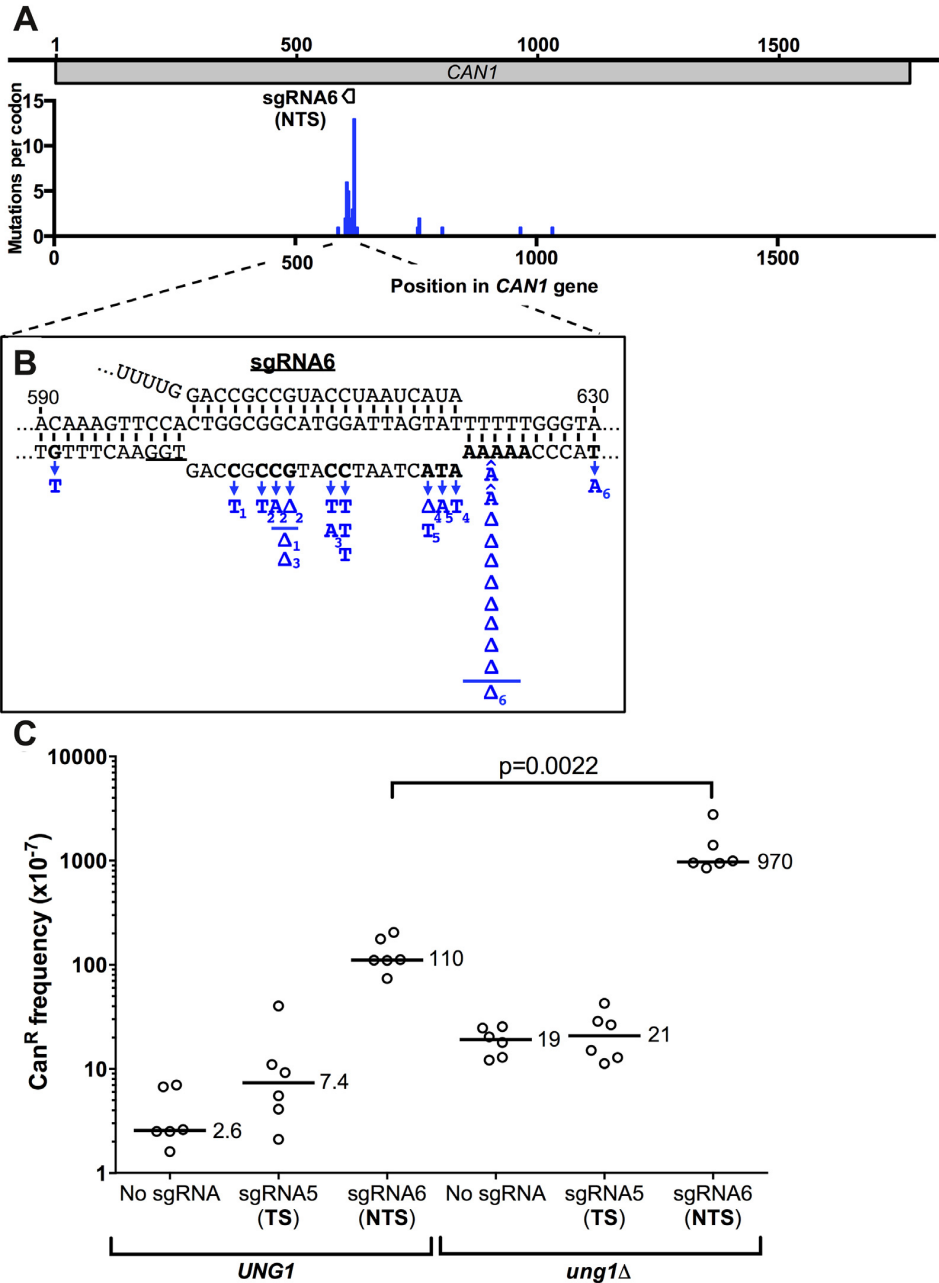
Can^R^ mutation spectrum for cells expressing dCas9 and sgRNA6 reveals a mutation hotspot at a homopolymer sequence in the guide RNA target. (**A**) Distribution of dCas9/sgRNA6-induced Can^R^ mutations. Mutations per *CAN1* codon is plotted. Location of sgRNA6 guide RNA target is depicted for reference. If multiple nucleotides are substituted or deleted, each mutated nucleotide is counted as individual mutation. (**B**) dCas9/sgRNA6-induced mutation spectrum. All mutations are indicated in blue, deletions are indicated with a Δ, and insertions are indicated with a ∧ immediately above the identity of the nucleotide inserted. For deletions that eliminate multiple nucleotides, a line above the deletion symbol indicates the extent of the deletion. Each of the mutations in a complex mutation event is indicated with the same subscript (i.e. T_1_Δ_1_). (**C**) dCas9/sgRNA6 induce mutations in part through a cytosine deamination mechanism. Frequency of canavanine-resistant (Can^R^) mutants in WT (*UNG1*) or *ung1*Δ mutant strains expressing dCas9 and the indicated guide RNA, as described in the Figure 1 legend. Data for *UNG1* wild-type strain is from Figure 1, and is included for reference. Statistical significance was calculated using a two-tailed Mann–Whitney U test.

Since sgRNA6 also targets a region of *CAN1* with a number of potential *CAN1*-inactivating cytosines (Supplementary Table S2), we tested whether this guide RNA also promoted cytosine deamination-based mutagenesis. Sequencing of Can^R^ isolates from dCas9/sgRNA6 expressing cells revealed that the *can1* mutants were clustered at the sgRNA6 target sequence, with 20 out of 25 sequenced isolates having a mutation in the sgRNA6 target site (Figure 6A). Closer inspection revealed that 4 of the sequenced isolates had a single nucleotide substitution in the guide RNA target, and all four of these were C>T mutations in the non-target strand (Figure 6B). Moreover, the frequency of Can^R^ mutants was significantly elevated when dCas9/sgRNA6 were expressed in an *ung1*Δ mutant strain (Figure 6C). These results indicate that dCas9/sgRNA6 promote *CAN1* mutagenesis in part through a cytosine deamination mechanism.

However, our sequencing data indicated that dCas9/sgRNA6 also induced many complex mutations, as well as insertion or deletion events, in the sgRNA6 target sequence (Figure 6B and Supplementary Table S1). Six out of the 20 target site mutations were complex mutations, suggesting that these complex events may be pol ζ-dependent. Notably, 10 out of 20 target site mutations (50%) were single nucleotide insertion or deletion events occurring at a 6-nucleotide homopolymer sequence that overlapped with the sgRNA6 target (Figure 6B). These data suggest that dCas9 binding may also promote mutagenesis when targeted to homopolymer repeats.

### Targeting of dCas9 by a mismatch-containing guide RNA also stimulates *CAN1* mutagenesis

**Figure 7.**
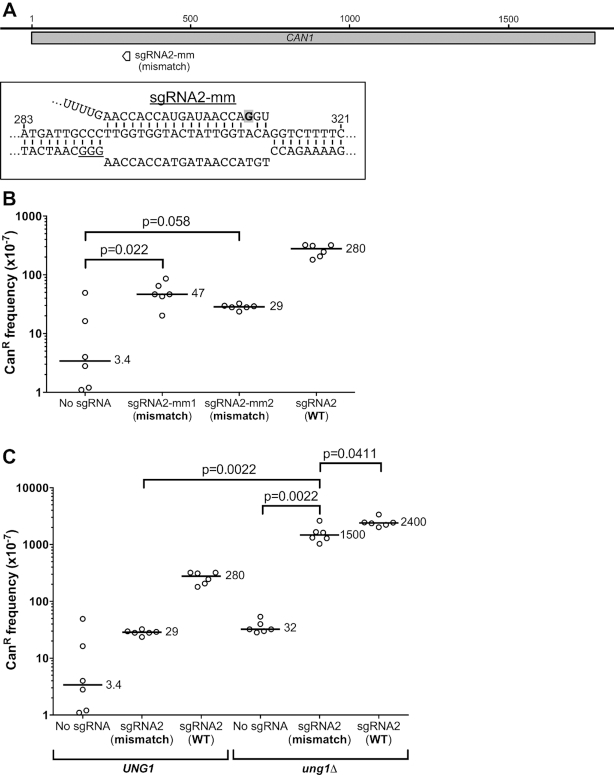
Targeting of dCas9 to the NTS of *CAN1* with a mismatch-containing guide RNA still induces mutagenesis, particularly in an *ung1*Δ mutant background. (**A**) Schematic showing the target site and mismatch location (gray shading) for the sgRNA2-mismatch (sgRNA2-mm) guide RNA. (**B**) Frequency of Can^R^ mutants in WT yeast expressing dCas9 and the indicated guide RNAs. sgRNA2-mm1 and sgRNA2-mm2 were two independent isolates of the mismatch sgRNA expressing plasmid that were assayed separately. Data for sgRNA2 (WT, indicating no mismatch) from Figure 1 is included for reference. Statistical significance was calculated using a two-tailed Mann–Whitney U test. (**C**) Same as a panel B, except the Can^R^ frequency was measured in WT or *ung1*Δ mutant strain. Data for *UNG1* WT background is from panel B, and data for sgRNA2 in the *ung1*Δ strain is from Figure 3, which are included for reference.

It is known that Cas9 (and dCas9) frequently bind off-target sites that contain one or more mismatches between the guide RNA and the DNA target. To determine whether dCas9 binding to off-target sites is mutagenic, we measured the frequency of Can^R^ mutations in a yeast strain expressing dCas9 and an sgRNA2 variant containing a mismatch with the DNA target 18 nt upstream of the PAM sequence (sgRNA2-mm, see Figure 7A). The frequency of Can^R^ mutants was consistently elevated in two independent isolates expressing an sgRNA2-mm guide with dCas9 relative to the ‘No sgRNA' control (∼14-fold and ∼9-fold in sgRNA2-mm1 and sgRNA2-mm2, respectively; see Figure 7B). However, the frequency of Can^R^ mutants was significantly reduced in the mismatch-containing sgRNAs relative to WT sgRNA2 (∼6- to ∼10-fold lower in Figure 7B, *P* = 0.0022), indicating that even a single mismatch between the guide RNA and the DNA target significantly reduces mutation frequency.

To test whether the mismatch-containing guide induced mutagenesis through a cytosine deamination mechanism, we measured the frequency of Can^R^ mutants in an *ung1*Δ mutant strain. The frequency of Can^R^ mutants was significantly elevated when dCas9 and sgRNA2-mm were expressed in the *ung1*Δ background relative to either the ‘No sgRNA' control or when dCas9 and sgRNA2-mm were expressed in an *UNG1* WT background (*P* = 0.0022 and 0.0022, respectively; see Figure 7C). Deletion of *ung1* in the dCas9 and sgRNA2-mm expressing strain increased the frequency of Can^R^ mutants by up to ∼50-fold (Figure 7C), consistent with the hypothesis that dCas9 targeted by mismatching-containing sgRNA induces mutations through a cytosine deamination mechanism, particularly in an *ung1*Δ mutant background. Moreover, in the *ung1*Δ mutant background, the mutation frequency induced by mismatch-containing sgRNA2 was only marginally different than the WT sgRNA2 (Figure 7C), suggesting that Ung1 repair activity may in some cases suppress mutagenesis at dCas9-bound off-target sites.

## DISCUSSION

**Figure 8.**
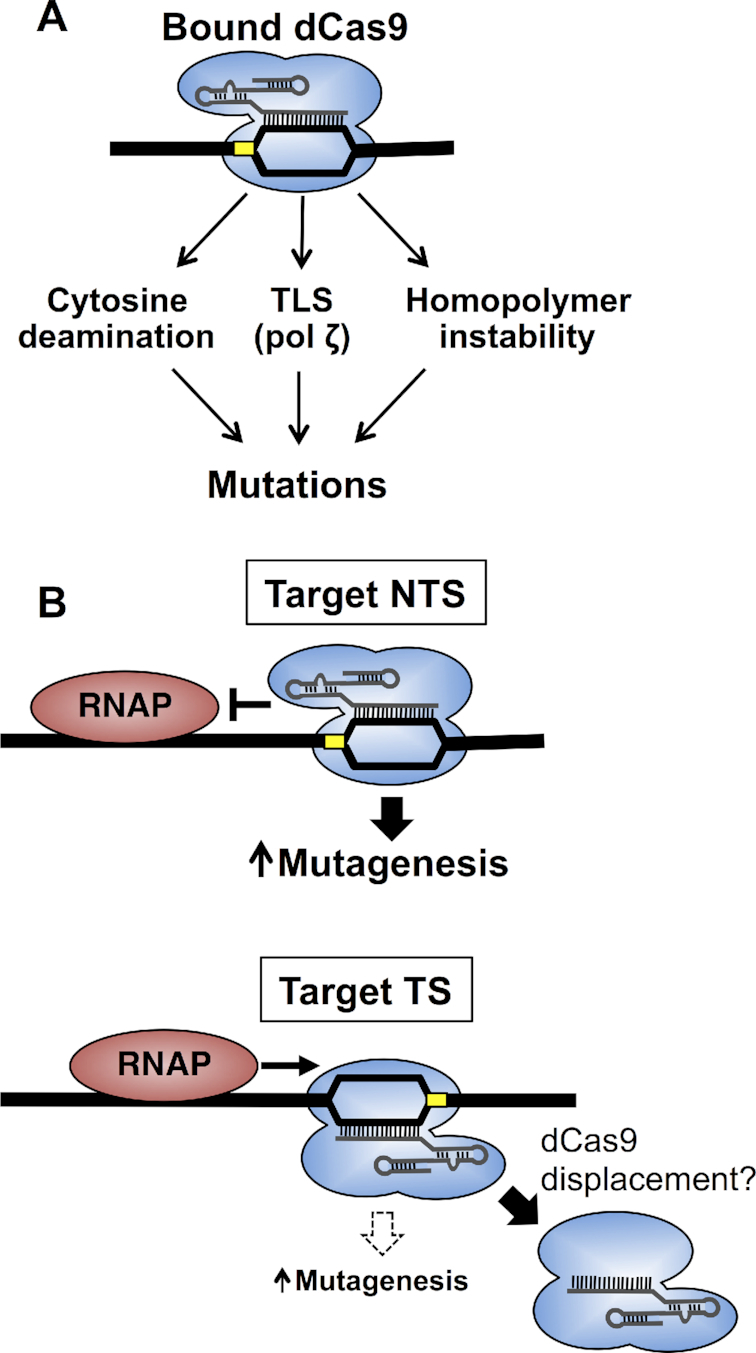
Model of how dCas9 binding induces mutations. (**A**) Our data indicate that the dCas9-induced R-loop promotes mutagenesis by at least three different mechanisms: cytosine deamination (in the non-target strand), promoting translesion DNA synthesis by DNA polymerase zeta (pol ζ), and causing homopolymer repeat instability. (**B**) We propose that guide RNAs targeted to the NTS are more mutagenic in yeast because dCas9 binding and R-loop formation is more persistent, since dCas9 binding to the NTS is more resistant to being displaced by RNA polymerase. In contrast, dCas9 targeting to the TS is likely more labile, and therefore less mutagenic, due to displacement by elongating RNA polymerase. The PAM site is indicated with a yellow box.

While it is well established that Cas9-mediated DNA cleavage is highly mutagenic in eukaryotic cells, it is generally assumed that DNA binding by Cas9 (or dCas9) does not affect mutation rates. Here, we show that DNA binding by dCas9 is mutagenic in yeast, particularly when dCas9 is targeted to the non-transcribed strand of a yeast gene. Multiple lines of evidence indicate that many dCas9-induced mutations arise in part due to cytosine deamination on the non-target DNA strand, which adopts a single-stranded DNA conformation during dCas9-induced R-loop formation. Moreover, we show that DNA binding by dCas9 is mutagenic even at mismatch-containing off-target sites, especially in cells lacking uracil glycosylase activity. However, dCas9 binding also can induce complex mutation events and cause instability at homopolymer sequences, indicating that dCas9 binding can induce mutations through multiple mechanisms in eukaryotic cells (Figure 8A).

We show that Can^R^ mutations due to dCas9 binding occur at a frequency of roughly ∼1 × 10^−5^ in yeast when targeting the NTS of *CAN1*. This mutation frequency is much lower than the reported mutation frequencies of 10^−2^–10^−3^ when endonuclease active Cas9 is targeted to *CAN1* (14,32). Presumably, this is the reason dCas9-induced mutations have not been previously detected in experiments with mammalian cells (e.g., (23)), since these studies may not have been sufficiently sensitive to detect such mutation events. One study in yeast did measure the dCas9-induced *CAN1* mutation frequency when targeted with a guide RNA equivalent to sgRNA4 (14), and reported a *CAN1* mutation frequency of ∼10^−5^–10^−6^ following targeting with dCas9 for 5 generations, which is roughly consistent with our data for dCas9/sgRNA4 (Figure 1). However, since these experiments lacked a ‘No sgRNA' control (i.e. the dCas9 experiment was the control), it was apparently not recognized that dCas9 binding alone affects mutation rates. Our results indicate that in the absence of uracil glycosylase activity (i.e., *ung1*Δ), dCas9-induced mutations occur at a frequency of ∼1 × 10^−4^ (or higher), a frequency that could be readily measured using mammalian mutational reporter assays.

A unique aspect of dCas9-induced mutagenesis is that mutation rates are highest when the guide RNAs is targeted to the non-transcribed strand. While we show that this is in part influenced by the number of potential *CAN1*-inactivating cytosines in the non-target strand of the guide RNA, our data suggest that guide RNAs targeting the NTS generally have a higher mutation frequency, even after accounting for the number of mutable cytosines in the guide RNA target. Although the underlying molecular mechanism is currently unclear, the simplest explanation is that RNA polymerase II efficiently displaces dCas9 when it targets the TS, but much less efficiently when dCas9 targets the NTS (Figure 8B). A previous study in *E. coli* has shown that dCas9 blocks RNA polymerase transcription when targeting the NTS, apparently because the RNA polymerase is unable to displace dCas9 when the PAM motif is oriented toward the elongating RNA polymerase (37). In contrast, RNA polymerase can transcribe through (and presumably displace) dCas9 when it targets the TS. This model would suggest that RNA polymerase II transcribing the *CAN1* gene would elongate through and displace dCas9 targeting the TS, thus limiting the residence time of the dCas9-induced R-loop, and thereby potentially mitigating the mutagenic effects of dCas9 binding in this context. In contrast, dCas9 targeting the NTS would remain bound to its DNA target to a greater extent, thereby more efficiently promoting mutagenesis (Figure 8B). Alternatively, dCas9 binding to the NTS could promote mutagenesis through other mechanisms, such as by promoting RNA polymerase stalling and/or transcription-coupled repair, or transcription-replication conflicts.

Complete inhibition of *CAN1* transcription by dCas9 can cause a Can^R^ phenotype in the absence of a *CAN1*-inactivating mutation (data not shown), and in our experiments we observed some background growth on canavanine-containing plates (e.g. for dCas9/sgRNA2 expressing cells). However, three lines of evidence suggest that *CAN1* transcriptional inhibition does not directly cause canavanine resistance in our experiments. First, constitutive expression of pGAL-dCas9 on galactose-containing canavanine plates does not induce canavanine resistance in sgRNA2 expressing cells (Supplementary Figure S3). Second, additional testing of a subset of Can^R^ colonies from a dCas9-sgRNA2 expressing strain confirmed that Can^R^ phenotype was independent of ongoing guide RNA expression, as the colonies retained a Can^R^ phenotype even after the sgRNA2-expressing plasmid was shuffled out by 5-fluoroorotic acid (FOA) selection (data not shown). Third, all Can^R^ colonies that have been sequenced contained a *CAN1*-inactivating mutation, which are frequently clustered near the guide RNA target site. In summary, these data confirm that dCas9 binding induces *CAN1* mutations, particularly near the guide RNA target site.

Our data indicate that dCas9 binding can induce mutations through a cytosine deamination-based mechanism. Cytosine deamination rates at the sgRNA target are likely elevated by R-loop formation during dCas9 binding. It has been postulated that R-loop formation may stimulate cytosine deamination (26,27), since the deamination rate is greatly elevated (>100-fold) in ssDNA (30). This is consistent with our finding that mutations likely due to cytosine deamination occur primarily on the non-target strand, which is single-stranded in the dCas9 R-loop. We hypothesize that cytosine deamination occurs spontaneously on the non-target strand in the dCas9 R-loop, since deletion of *FCY1*, the only reported cytidine deaminase in yeast (41), did not affect the frequency of dCas9-induced Can^R^ mutants. Moreover, the location of dCas9-induced mutations within the guide RNA target is inconsistent with a cytidine deaminase mechanism, since many of the mutations occur within the PAM-proximal ‘seed' region (see Figures 2C, 3D, and 5C). This region is normally not targeted by cytidine deaminases (13,14) because it is located in an inaccessible tunnel in the dCas9 protein (19,46). An intriguing possibility is that the chemical environment within this tunnel might alter the rate of spontaneous cytosine deamination in the non-target DNA strand.

While cytosine deamination clearly plays an important role in dCas9-induced mutagenesis, particularly for certain guide RNAs (e.g. sgRNA2, sgRNA6 and sgRNA7), other guide RNAs appear to primarily induce mutations through alternate mechanisms (Figure 8A). For example, the dCas9/sgRNA1 mutation spectrum is enriched for complex mutations near the guide RNA target, which we show are dependent upon TLS activity by pol ζ. Other guide RNAs (e.g., sgRNA6 and sgRNA7) also had a relatively high number of complex mutations near their DNA targets, indicating that pol ζ-mediated complex mutation events are likely a common mechanism for dCas9 mutagenesis. We speculate that dCas9 binding may induce DNA polymerase stalling, which could lead to recruitment of TLS polymerases such as Pol ζ. We also observed a very high frequency of single nucleotide insertion/deletion events at a 6-nucleotide homopolymer sequence targeted by sgRNA6. Insertion/deletion events at homopolymer sequences can arise due to DNA polymerase slippage and template misalignment at such sequences during replication (47,48). Such events are typically corrected by mismatch repair, as defects in mismatch repair cause an elevated frequency of insertion and deletion mutations at homopolymers and other repeat sequences (49–52). We hypothesize that dCas9 binding to a homopolymer sequence could promote DNA polymerase slippage and/or inhibit subsequent mismatch repair, leading to an increased frequency of insertion and deletion mutations. Future experiments are required to elucidate which of these mechanisms contributes to dCas9 mutagenesis at homopolymer sequences.

An important implication of these findings is that many popular dCas9 applications, including transcriptional activation, repression (3–8), or epigenome editing (9–11), may also be causing unwanted mutagenesis at the dCas9 target site. Our results indicate that dCas9 binding promotes mutagenesis even at off-target binding sites. In WT cells, the dCas9-induced mutation frequency is reduced by the presence of mismatches between the sgRNA and DNA target; however, in an *ung1*Δ mutant strain, dCas9-induced mutagenesis was greatly elevated at mismatch-containing targets and approached the mutation frequencies observed in WT cells. It will be important in future studies to determine whether dCas9 binding to mismatch-containing off-target sites can also stimulate pol ζ-mediated complex mutation events and homopolymer repeat instability.

In summary, our data indicate that dCas9 targeting promotes mutagenesis in yeast, likely due to the mutagenic effects of dCas9-mediated R-loop formation. These findings not only have important implications for background mutagenesis in dCas9 (and Cas9) applications, but also provide a novel method for studying how targeted R-loop formation promotes genome instability and mutagenesis in eukaryotic cells.

## Supplementary Material

Supplementary Data
