# Supplementary material for: R-loop formation by dCas9 is mutagenic in Saccharomyces cerevisiae
Source: Nucleic Acids Res. 2018 Dec 21;47(5):2389–401. doi: 10.1093/nar/gky1278 (PMC6411842; doi:10.1093/nar/gky1278)
Supplement: Supplementary Data [file gky1278_supplemental_files.zip › Laughery_etal_SuppMaterials_revision_finalJW.pdf]

SUPPLEMENTAL MATERIALS

[Note: Supplemental Table S1 is in a separate file.]

Supplemental Table S2: Number of Can1-inactivating cytosines in the non-target strand of the guide RNA targets

Guide RNA	*Location of Target*[a]	*Number of Can1-inactivating cytosines in non-target strand*[b]	*Positions of Can1-inactivating cytosines*[c]
sgRNA1 (TS)	277-296 in *CAN1*	0	–
sgRNA2 (NTS)	293-312 in *CAN1*	5	G295, G298, G299, G307, G308
sgRNA3 (TS)	754-773 in *CAN1*	1	C755
sgRNA4 (NTS)	767-786 in *CAN1*	2	G775, G776
sgRNA5 (TS)	590-609 in *CAN1*	0	–
sgRNA6 (NTS)	601-620 in *CAN1*	2	G611, G612
New Guides			
sgRNA7 (TS)	399-418 in *CAN1*	3	C404, C412, C416
sgRNA8 (TS)	408-427 in *CAN1*	2	C412, C416

[a]Indicates the nucleotide position of the R-loop forming target site in *CAN1* (not including PAM).
[b]Can1-inactivating cytosines are cytosines which when mutated to a different base are associated with a Can^R phenotype. For NTS guide RNAs, this would be the number of Can1-inactivating guanines, which are cytosines on the non-target strand. Data derived from analysis of references 36 and 45 (see main manuscript text). Cytosine mutations that cause a silent mutation were excluded.
[c]For NTS guides, non-target strand cytosines are guanines in the *CAN1* coding sequence.

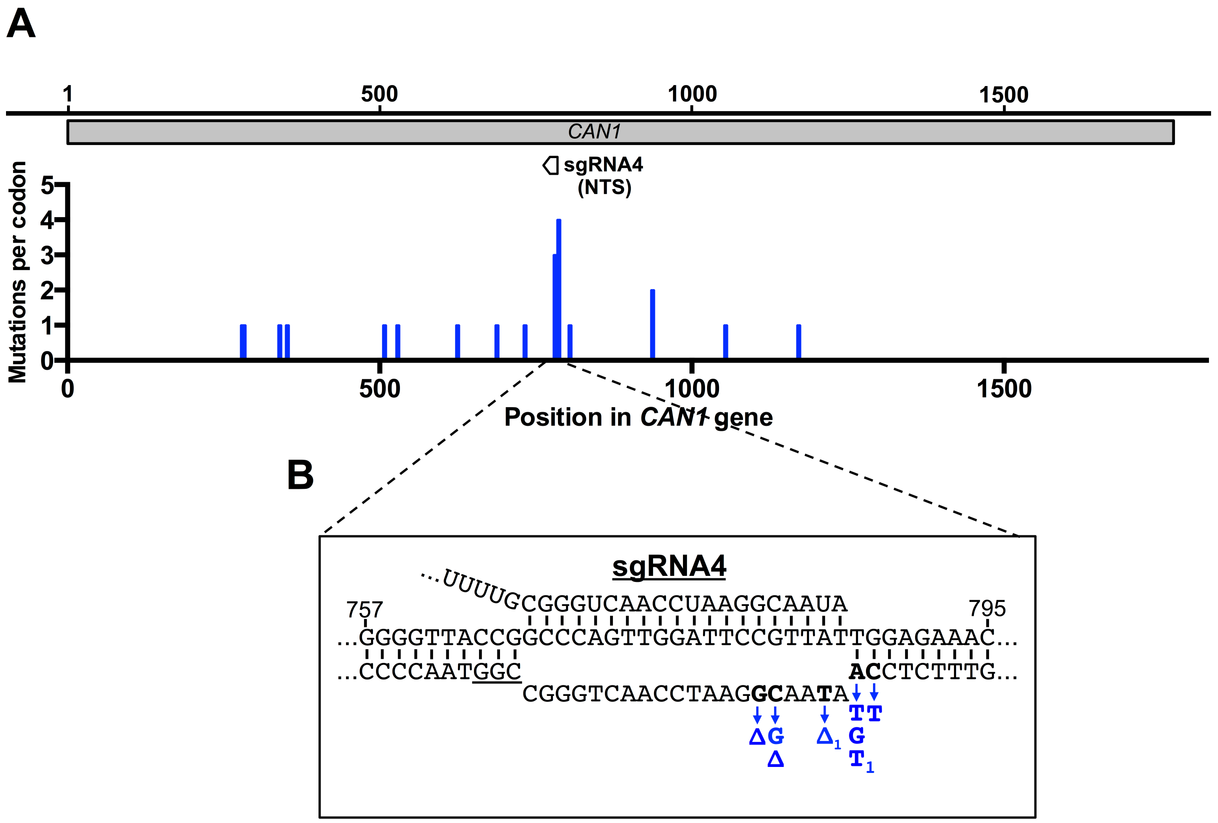

Supplemental Figure S1. dCas9-induced *can1* mutations are clustered at the guide RNA target. (A) Distribution of Can^{R} mutations in WT yeast expressing dCas9 and sgRNA4, which targets the *CAN1* non-transcribed strand (NTS). (B) dCas9/sgRNA4-induced substitution mutations or deletions (Δ) are indicated in blue. A tandem mutation in the same mutant isolate ($T_{785}\Delta$ and A_{787}->T on the non-target strand) is indicated with a subscript 1.

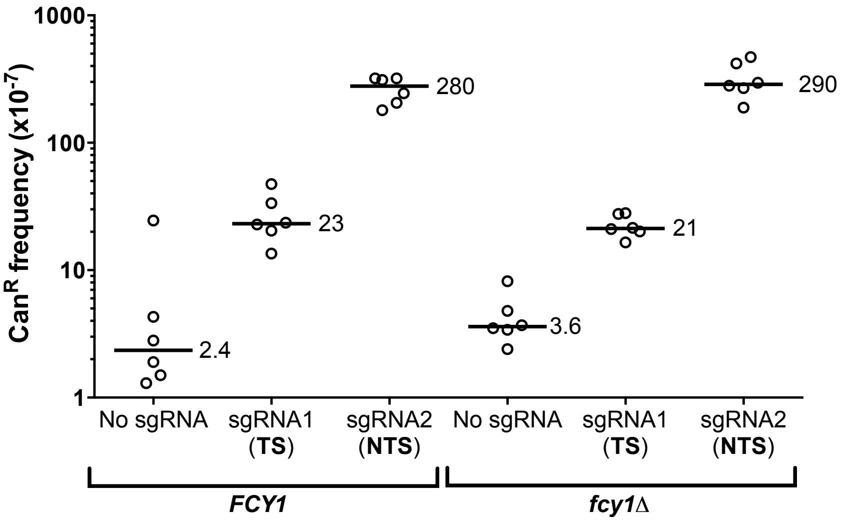

Supplemental Figure S2. Yeast Fcy1 cytosine deaminase is not required for dCas9-induced mutagenesis. Frequency of canavanine resistant (Can^R) mutants in WT (*FCY1*) or *fcy1*Δ mutant strains expressing dCas9 and the indicated guide RNA, as described in the Figure 1 legend. Data for *FCY1* wild-type strain is from Figure 1, and is included for reference.

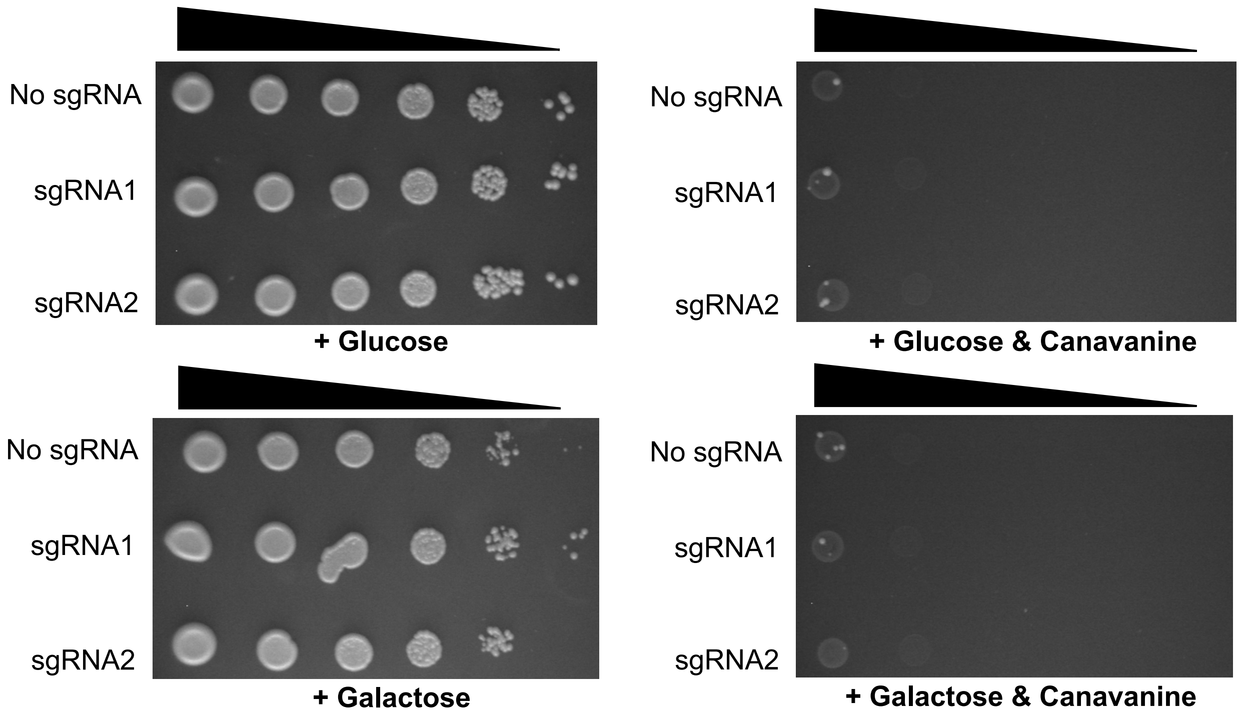

Supplemental Figure S3. Yeast cells expressing dCas9 under the control of GAL promoter (pGAL-dCas9) and a second vector encoding sgRNA1, sgRNA2, or no sgRNA were spotted on plates containing SC+ 2% Glucose (top left), SC-Arg + 2% Glucose + 0.006% Canavanine (top right), SC+ 2% Galactose (bottom left), and SC-Arg + 2% Galactose + 0.006% Canavanine (bottom right).
